# Neuroleptic malignant-like syndrome associated multiple system atrophy: report on three cases

**DOI:** 10.1186/s12883-022-02583-8

**Published:** 2022-02-25

**Authors:** Yan Lin, Lin Ma, Nan Zhang, Ruihua Li, Wenjing Jiang

**Affiliations:** 1 Department of Geriatric Medicine and Shandong Key Laboratory of Cardiovascular Proteomics, Qilu Hospital, Cheeloo College of Medicine, Shandong University, No. 107 West Wenhua Road, Jinan, 250012 Shandong China; 2 Shandong Key Laboratory of Cardiovascular Proteomics, Qilu Hospital, Shandong University, Jinan, 250012 Shandong China

**Keywords:** Neuroleptic malignant-like syndrome, Multiple system atrophy, Dopaminergic medication, Hyperthermia

## Abstract

**Background:**

Multiple system atrophy (MSA) associated with neuroleptic malignant-like syndrome (NMLS) is rare and few cases have been described in the literature.

**Case presentation:**

In the present study, three patients with MSA associated with NMLS were analyzed from January 2012 to January 2020 to characterize their clinical presentations. Data collected from the patients for analysis included general patient history, the fluctuation and severity of disease symptoms, the indicated therapies and disease progression at follow-up. All patients had histories of sudden withdrawal or reduction of levodopa prior to the onset of symptoms. Clinical presentations were characterized by hyperthermia, autonomic dysfunction, worsening of extrapyramidal symptoms, and elevated serum creatine kinase (CK) levels. During hospitalization, one patient rapidly progressed and died, while the other two patients were successfully treated.

**Conclusions:**

Early diagnosis and treatment are very important for patient outcomes in NMLS. Notably, the correct dose and time of administration of dopaminergic medication may be key in treating NMLS.

**Supplementary Information:**

The online version contains supplementary material available at 10.1186/s12883-022-02583-8.

## Background

In 1968, Delay et al. described the first case of Neuroleptic Malignant Syndrome (NMS), a potentially fatal condition characterized by hyperthermia, altered consciousness (agitation, delirium, or coma), autonomic dysfunction, extrapyramidal symptoms, muscle cramps or tremors, and elevated serum creatine kinase (CK) levels [1]. Although disturbances in the brain’s monoaminergic systems or rapid withdrawal of drugs for Parkinson’s disease may cause the condition, it is most frequently observed in patients receiving treatment with neuroleptic drugs [2, 3].

In 1981, Toru M. et al., reported a rare complication of sudden withdrawal or reduction of anti-parkinsonian treatment for Parkinson’s disease (PD), as well as of abrupt switching from one agent to another [4]. This condition was called parkinsonism hyperpyrexia syndrome or more commonly referred to as Neuroleptic malignant-like syndrome (NMLS). Clinical manifestations of NMLS are very similar to NMS, including hyperthermia, mental status change, muscular rigidity, respiratory failure, and autonomic instability [5, 6]. Like NMS, the pathophysiological mechanism of NMLS is still unclear. Due to the acute dopamine-deficient state frequently associated with NMLS, most hypotheses of its pathogenesis suspect dopamine receptor blockade and dysfunction of the dopamine receptor D2 [3, 7]. Published case series also speculate that other contributing factors including α-synuclein and ubiquitin-related diseases (Lewy body dementia and some lysosomal storage diseases), dehydration, physical exhaustion, and acute structural or functional brain disorders (including encephalitis, tumor, surgery, and trauma) are involved in NMLS [8–11].

Another ubiquitin and α-synuclein-related disease is multiple system atrophy (MSA). Its most common manifestation is autonomic dysfunction. However, its other signs and symptoms are mainly divided into two subtypes: parkinsonian manifestations (rigidity, tremors, bradykinesia, and postural instability) and cerebellar manifestation (unsteady gait, dysarthria, difficulty swallowing and double vision) [12]. The disease is termed MSA-P when there is a preponderance of parkinsonian symptoms and MSA-C when cerebellar symptoms are more dominant [12–14]. Previous studies have described catecholaminergic agents as potential therapies for MSA.

In our present study, we report three MSA patients who presented with NMLS. We analyzed their clinical features and summarized the therapeutic processes undertaken. Our aim is to improve awareness and effective treatment of this disease among clinicians.

## Case presentation

Three cases of MSA associated-NMLS were identified in the Department of Geriatrics and Neurology (Qilu Hospital of Shandong University, Jinan, China) between 2012 and 2020. All three patients were diagnosed with MSA according to the clinical diagnostic criteria updated in 2008 [13]. The score for probability of NMLS consists of 8 symptoms based on international expert consensus (IEC) diagnostic criteria [15] (Supplementary Table 1). Shandong University’s Institutional Review Board ethics committee (Qilu Hospital, Jinan, China) reviewed and approved the study. All patients provided written informed consent prior to inclusion in the study.

### Patient 1

**Fig. 1:**
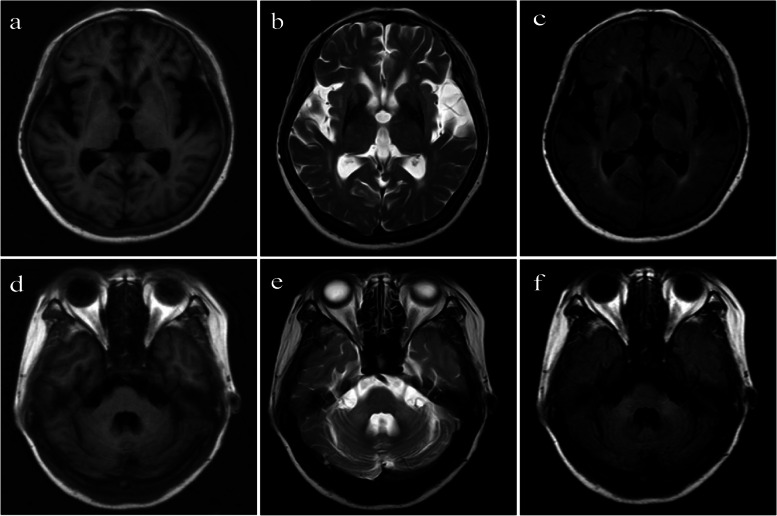
MRI showed atrophy of the brainstem and cerebellum, enlarged bilateral ventricle, iron deposition in the bilateral putamen, and a hyperintense putaminal rim. **a** and **d** Contrast-enhanced T1-weighted image. **b** and **e** Contrast-enhanced T2-weighted image. **c** and **f** T2/FLAIR image. MRI: Magnetic resonance imaging; FLAIR: Fluid attenuation inversion recovery

In Nov 2012, a female patient aged 62 was diagnosed as having probable MSA. She had presented with a syndrome of muscular rigidity and ataxia for 2 years. In addition, she had also developed glossolalia, vegetative nerve functional disturbance, sebaceous gland secretions, alternating high and low body temperatures, incontinence, and constipation over 6 months. Brain MRI suggested a diagnosis of MSA, which showed atrophy of the brainstem and cerebellum, enlarged lateral ventricles, putaminal iron deposition, and a hyperintense putaminal rim (Fig. 1). The patient had been on levodopa (480 mg/day), comprised of Madopar (175 mg, twice a day) and Sinemet (250 mg, at night), but her clinical syndromes gradually deteriorated. The patient gradually tapered down medications over 11 months and, by October 2013, had discontinued all dopaminergic drugs.

On November 25 in 2013, she was hospitalized for hyperthermia. Initial investigations showed her blood pressure to be in the normal range (125/61 mmHg), while her body temperature was raised to 39.3 °C with the whole-body sweating. On admission, hematologic tests showed a raised leukocyte count of 6.81 × 10^9^/L (normal: 3.5–9.5 × 10^9^/L), a slightly raised CK of 472 U/L (normal: 26–178 U/L), and hyponatremia (sodium, 125 mmol/L, normal range 137–147 U/L). The admission sputum, urine, and blood cultures were all negative, eliminating the presence of intercurrent infections. Both serum levels of tumor markers (including:α-fetoprotein, squamous cell carcinoma, and carcinoembryonic antigen) and autoimmune antibody tests were within the normal range, suggesting that malignant and immune-related diseases were also absent. Computer tomography (CT) of the chest, abdomen, and pelvic cavity showed coronary artery calcification and pulmonary fibrosis.

The patient’s symptoms progressed to laryngospasm, shortness of breath, and rigidity on the second day following hospital admission. The respiratory rate ranged from 25 to 30 beats/min and the pulse rate was 120 beats/min. Analysis of blood gases from the patient’s arteries revealed a pH of 7.5 (normal: 7.35–7.45), indicating respiratory alkalosis. Moreover, the carbon dioxide and oxygen partial pressures were 27 mmHg (pCO2, normal: 35–45 mmHg) and 93 mmHg (pO2, normal: 80–100 mmHg) respectively. Blood results also showed that the lactic acid was 4.7 mmol/L (0.5–1.8 mmol/L), the total carbon dioxide was 21.9 (22–29 mmol/L), the standard base excess was − 2.1 mmol/L, and the actual base excess was − 0.8 mmol/L (− 2–3 mmol/L).

On day 3, her temperature rose to 39.3 °C which led to a working diagnosis of NMLS at the time. The patient’s levodopa dosage was therefore raised to 680 mg/day. The CK decreased to 252 U/L on the fourth day while the leukocyte count was still in the normal range. After increasing the dose of levodopa for 3 days, the patient’s temperature decreased to 37.5 °C. In tandem, the CK level also fell to 212 U/L. Two days later, the temperature was normal without any changes in therapy. This patient showed good health without recurrence over 5-year follow up.

### Patient 2

A female patient aged 63 with a history of bradykinesia and dysautonomia spanning 6 years was also examined. In 2013, she had developed glossolalia. Although she suffered serious ataxia with dystonia, her movements were voluntary. She was diagnosed as possible MSA in 2014. In the subsequent 2 years, she gradually started experiencing tremor, constipation, and retention of urine. The symptoms progressively worsened resulting her being unable to walk unaided.

On June 25, 2016, the patient complained of low-grade fever, and was admitted to our hospital. She had been on levodopa 800 mg/day, including 250 mg Sinemet four times a day, and had 50 mg piribedil twice a day, but showed poor response to these medications. Due to the poor response, the patient was withdrawn these drugs over 2 months. On admission, her body temperature was 37.8 °C. The initial blood examination showed that the leukocyte count was 6.51 × 10^9^/L, the serum level of sodium was 129 mmol/L, and the CK level was normal. Over the subsequent 3 days, she suffered pyrexia with a temperature of 40.9 °C, whole-body sweating and rigidity. The CK level rose gradually from 210 U/L to 976 U/L. Admission cultures of blood, sputum, and urine were negative, and intercurrent infections were ruled out. Results of tests for rheumatic, neoplastic, and immune markers, as well as thyroid hormones were within normal ranges. Empiric therapy was administered at all admissions. The antibiotics used ranged from third generation cephalosporins to Meropenem, but the temperature could not be controlled. On the fifth day she suddenly developed acute respiratory failure. Arterial blood gas analysis showed that the pO2 was 68 mmHg and the pCO2 was 32 mmHg. Although her breathing stabilized after endotracheal intubation, she finally died of disseminated intravascular coagulation (DIC) 1 day later.

### Patient 3

A male patient aged 78 presented with frozen gait, autonomic dysfunction, and parkinsonism over 10 years. Brain MRI demonstrated cortical subtentorial and cerebellar atrophy. He was diagnosed as probable MSA in 2016 because of the clinical features and imaging manifestations. After the diagnosis, he commenced taking anti-parkinsonian drugs. He had been kept in bed for 3 years and had not been able to communicate with family members for 2 years. He had thought that the drugs did not give much response, so had stopped all anti-parkinsonian drugs for the previous year. During the past month, he developed intermittent fever (sometimes up to 38.5 °C) which usually persisted for several days and then gradually resolved without any obvious cause.

Recently, a persistent fever resulted in his admission to our hospital. The patient manifested generalized muscular rigidity and sweating. Routine blood tests revealed his white blood cell count to be 8.86 × 10^9^/L, the serum level of sodium was 130 mmol/L, and the CK level was 418 U/L. The admission cultures of blood, sputum, and urine were negative. CT of his chest showed fibrosis at the base of the lungs. His progress worsened continuously after the administration of different levels of antibiotics. He was given levodopa at a dose of 300 mg/day (125 mg Madopar three times a day). This resulted in gradual control of his body temperature. The patient continued taking anti-parkinsonian drugs after discharge and the fever did not appear again.

## Discussion and conclusions

The prevalence of NMLS is high and it often requires rapid medical attention as it has a concomitantly high mortality rate. While mortality has reduced over the last few decades due to earlier diagnosis and aggressive intervention, the overall mortality of NMLS reported in the literature is still between 10 and 30% [16]. In the clinic, NMLS is sometimes referred to as parkinsonism hyperpyrexia syndrome. Manifestations usually include hyperthermia, altered consciousness, rigidity, sweating, fast heart rate, and autonomic imbalance [17, 18]. Other signs may include tachycardia, tachypnea, acidosis, incontinence, and elevated serum CK [18]. Accurate NMLS detection and therapy often relies upon its suspicion by a knowledgeable clinician rather than systematic diagnosis.

Previous studies have suggested that causes of NMLS include sudden withdrawal of anti-parkinsonian drugs, reduction or withdrawal of dopamine agonist therapy, and the substitution of anti-parkinsonian drugs with alternatives over a short space of time [18, 19]. Moreover, infection, trauma and surgery have also been implicated [2, 10]. Since dopaminergic drugs are also commonly used to treat MSA, in theory, NMLS conditions may also occur in patients with MSA.

Despite its low prevalence, MSA is associated with a variety of symptoms making it a progressive, degenerative neurological disease that is worthy of serious attention. Symptoms include general autonomic dysfunction (blood pressure, heart rate, sweating, and bladder function), parkinsonism symptoms, and cerebellar ataxia signs [12, 20]. MSA typically affects individuals in their 50s and 60s and the average survival following symptomatic onset is 6–10 years [21]. Causes of MSA are still unclear. Some scholars have proposed that an inherited component or environmental toxin may be responsible, but this remains controversial [22].

Microscopically, the brain tissue of patients shows neuronal destruction and contains abnormal filamentous α-synuclein aggregates in the glial cell and neuronal cytoplasm [12, 20, 23].

The present study reported on the clinical manifestations and laboratory examinations of three MSA cases associated with NMLS. Based on clinical experience, hyperthermia is recorded as an initial symptom [24]. In most cases, the temperatures exceed 38 °C. However, even higher temperatures above 40 °C can sometimes appear [18]. NMLS cases without hyperthermia have been rarely been reported [25]. The cause of hyperthermia in NMLS is still unclear. Some researchers have proposed that central dopamine receptor blockade in the hypothalamus impairs thermoregulation resulting in high fever [3]. In our study, all three patients presented the symptom of hyperthermia. Another common clinical feature of the three patients was hyponatremia. We have found only a few reports in the literature describing hyponatremia in patients with MSA or NMLS [26–28]. We speculate that hyponatremia was perhaps related to the feverish conditions experienced by the patients.

Severe rigidity of most body muscles is frequently seen at diagnosis. In parallel, the CK levels are almost always raised early in the progression of NMLS, indicating that CK may be a potential diagnostic marker for the condition [5, 17]. Generally CK elevation was mild to moderate and usually did not exceed 1000 U/L. CK levels, however, can remain normal if there is only mild muscle rigidity [29]. For our investigation, all three patients had normal or slightly increased CK levels. Other studies have reported that the onset of muscle rigidity and rise in CK level may not be simultaneous [30]. Indeed, the CK levels usually return to normal following an episode of NMLS.

Due to its mimicry of other syndromes, distinguishing NMLS from other neurological diseases in the early stages can be very difficult. As the possibility of NMLS is often overlooked and immediate treatment for the syndrome is delayed, expert judgment is required to differentiate it from other diseases. Initially, we did not recognize Patient 2 as having NMLS, which delayed the first treatment. Some of the most common misdiagnoses are encephalitis, toxic encephalopathy, status epilepticus, acute spinal cord injury, heat stroke, and malignant hyperthermia [31–33].

NMLS is a medical emergency that may rapidly lead to death if left untreated. The treatment for patients with NMLS should be based on three aspects: reinstating anti-parkinsonian drugs, supportive care, and specific treatments [16]. Doctors should remind MSA patients not to withdraw or reduce doses of anti-parkinsonian medications without a doctor’s supervision. Once NMLS appears, levodopa or dopamine agonists must be administered immediately [34]. In terms of supportive care, the following measures should be provided: cardiorespiratory function must be preserved (by using anti-arrhythmic drugs, mechanical ventilation, or pacemakers); euvolemia preservation with intravenous fluids; fever dissipation using cooling blankets; blood pressure reduction if markedly elevate and heparin or low-molecular weight heparin for the prevention of deep venous thrombosis. Dantrolene sodium as a muscle relaxant that can be used to reduced heat production and muscle rigidity [35]. Bromocriptine, Amantadine, Lorazepam, Phenytoin, Apomorphine, Aripiprazole, and anticholinergic agents have also been previously reported to be useful for NMLS [36–39].

Consideration of these patient cases will serve as a useful reminder that it is essential to consider NMLS in MSA. When a patient has a history of dopamine withdrawal and presents with two or more of the following primary symptoms: rigidity, mental status change, dysautonomia, or fever, NMLS should be suspected. The correct dose and timing of administration of dopaminergic medication is vital and should start as soon as possible.

## Supplementary Information

**Additional file 1.**

## Data Availability

All data related to this case report are documented within this manuscript.

## Abbreviations

MSA: Multiple system atrophy
NMLS: Neuroleptic malignant like syndrome
CK: Creatine kinase
NMS: Neuroleptic Malignant Syndrome
PD: Parkinson’s disease
CT: Computer tomography
DIC: Disseminated intravascular coagulation
