# Supplementary material for: Neuroleptic malignant-like syndrome associated multiple system atrophy: report on three cases
Source: BMC Neurol. 2022 Feb 25;22:67. doi: 10.1186/s12883-022-02583-8 (PMC8876065; doi:10.1186/s12883-022-02583-8)
Supplement: Supplementary file 1 — Additional file 1. [file 12883_2022_2583_MOESM1_ESM.docx]

**Supplementary Table 1. The score for probability of NMLS for patients.**

| **Diagnostic Criterion (8 symptoms)** | Patient 1 | Patient 2 | Patient 3 |
| --- | --- | --- | --- |
| Exposure to dopamine antagonist or dopamine agonist withdrawal, within the past 72 h | 0 | 0 | 0 |
| Hyperthermia (>100.4°F or >38.0°C on at least 2 occasions, measured orally) | 18 | 18 | 18 |
| Rigidity | 17 | 17 | 17 |
| Mental status alteration (reduced or fluctuating level of consciousness) | 13 | 13 | 13 |
| Creatine kinase elevation (at least 4 times upper limit of normal) | 0 | 10 | 0 |
| Sympathetic nervous system lability, defined as at least 2 of the following:  Blood pressure elevation (systolic or diastolic≥25% above baseline)  Blood pressure fluctuation (≥20 mm Hg diastolic change or ≥25 mm Hg systolic change within 24h  Diaphoresis  Urinary incontinence | 10 | 10 | 10 |
| Hypermetabolsim, defined as heart rate increase (≥25% above baseline) and respiratory rate increase (≥50% above baseline) | 5 | 5 | 5 |
| Negative workup for infectious, toxic, metabolic, and neurologic causes | 7 | 7 | 7 |
| Total | 70 | 80 | 70 |
